# Authors’ reply to letter by Martinod *et al.* on rationale for using lenvatinib as an urgent initial therapy in thyroid cancer with remarkable laryngotracheal invasion

**DOI:** 10.1530/ETJ-25-0377

**Published:** 2025-12-24

**Authors:** Hiroshi Katoh, Riku Okamoto, Yuka Ozawa, Takaaki Tokito, Mariko Kikuchi, Takafumi Sangai

**Affiliations:** Department of Breast and Thyroid Surgery, Kitasato University Hospital, Sagamihara, Kanagawa, Japan

**Keywords:** tracheal invasion, laryngeal invasion, lenvatinib, turn-around-time, thyroid cancer

Dear Editor,

We sincerely appreciate the thoughtful and constructive comments provided by Martinod *et al.* (1) regarding our article (2). Their letter highlights important aspects of the management of thyroid cancers with laryngotracheal invasion. We address each of the concerns below and clarify several points that may have been misunderstood.

## Regarding tracheal or laryngotracheal replacement surgery

We fully acknowledge that advanced surgical techniques, including tracheal or laryngotracheal replacement using stented aortic matrices reported in the TRITON-01 registry (3, 4, 5), represent promising options for selected patients. However, our study focused on patients presenting with life-threatening airway stenosis requiring urgent intervention. In Japan, such highly specialized airway replacement surgery is currently available only at a limited number of institutions, and when patients are transferred in acute settings with critical stenosis, immediate reconstructive surgery is often not feasible as the initial step. Our aim was not to compare lenvatinib (LEN) therapy with advanced reconstructive surgery, but rather to document that rapid tumor shrinkage induced by LEN can stabilize the airway and avoid emergency invasive procedures – such as tracheostomy or airway stenting – in real-world urgent situations.

## Regarding the inclusion of various histologies and the focus on anaplastic thyroid cancer (ATC)

It is correct that 10 out of 14 patients in our cohort had ATC. However, the purpose of our study was to evaluate urgent initial or bridging therapy for ‘remarkable laryngotracheal invasion’, independent of histology, because the immediate clinical problem in these patients is airway compromise rather than long-term oncologic outcome.

The authors pointed out that most of our patients did not undergo surgery; however, we would like to clarify that conversion surgery was performed in one poorly differentiated thyroid cancer (PDTC) and three ATC cases (two ATC cases at the time of original manuscript submission). In addition, one patient with papillary thyroid carcinoma became eligible for curative surgery, and this option was presented to the patient. Nevertheless, because she has now reached the age of 84, has maintained stable disease on low-dose LEN without any significant adverse events, and was personally reluctant to undergo surgery, she ultimately declined operative treatment. As noted in the Discussion section, we also consider that in very elderly patients – particularly those in their mid-80s or older – even differentiated thyroid carcinoma with laryngotracheal invasion may warrant prioritizing a rapid-acting, less invasive therapeutic option such as LEN rather than highly aggressive interventions.

We agree that ATC constitutes the major population in which such urgent measures are required. We have clarified in the manuscript that the findings mainly apply to ATC, and we appreciate the opportunity to further emphasize this point.

## Regarding long-term oncologic outcomes

The focus of our study was specifically the acute phase, documenting rapid symptom relief and improvement in airway diameter. We intentionally did not draw conclusions about long-term disease control because LEN was not intended as definitive therapy in this context. Our aim was to highlight that short-term airway stabilization enables patients to proceed safely to subsequent standard treatments – including surgery, targeted therapies, or radiation – once the full evaluation is completed.

## Regarding the use of lenvatinib for ATC

We agree that the ATA, NCCN, and TUTHYREF guidelines do not list LEN monotherapy as a treatment option for ATC. However, guideline status and reimbursement conditions differ by country: in Japan, LEN monotherapy remains formally approved and reimbursed for ATC, whereas combination therapy of LEN with pembrolizumab is not yet reimbursed (6). This major difference in regulatory and reimbursement circumstances explains why our study evaluated LEN monotherapy, which is currently the limited systemic therapy accessible immediately in emergency settings for ATC, as well as paclitaxel, in Japan.

Furthermore, although the Japanese phase II HOPE trial did not demonstrate an overall survival benefit in ATC, it did show a disease control rate of 73.8% (7), indicating that LEN does have tumor-controlling effects and can therefore serve as a viable bridging therapy in ATC.

Our paper does not claim LEN monotherapy as a definitive first-line regimen for unresectable ATC, but rather reports its utility as an urgent initial intervention for airway management.

## Regarding molecular testing turnaround time (TAT)

We recognize that in some expert centers – including the authors’ institution – digital PCR enables mutation profiling within 24 h. However, this situation does not reflect the reality at least in most Japanese clinical settings: companion diagnostic tests approved for reimbursement must be used to prescribe mutation-matched targeted therapies. Even centers capable of rapid in-house testing cannot bill these analyses, resulting in significant institutional cost burdens. Consequently, many hospitals rely exclusively on approved companion diagnostics, leading to a TAT of approximately 1 week before targeted agents can be initiated.

Thus, contrary to the authors’ assumptions, a clinically meaningful TAT still exists in many institutions, at least in Japan, and a safe bridging therapy is often necessary – particularly for patients presenting with critical airway obstruction – during the waiting period required for molecular testing. Our findings demonstrate that LEN can effectively maintain airway patency during this unavoidable interval.

In conclusion, we appreciate the opportunity to clarify the context and intent of our study. While we agree that emerging surgical techniques and rapid molecular testing are highly valuable, their availability differs across countries and institutions at present. Our study reflects the real-world clinical constraints in Japan, where LEN monotherapy remains the limited, readily accessible systemic option during urgent airway compromise in ATC.

We hope that our reply appropriately contextualizes our findings and contributes to constructive discussion on the management of thyroid cancers with severe laryngotracheal invasion.

## Declaration of interest

The authors declare that there is no conflict of interest that could be perceived as prejudicing the impartiality of the work reported.

## Funding

This work did not receive any specific grant from any funding agency in the public, commercial, or not-for-profit sector.
